# Perspectives on Barriers to Eating Healthy Among Food Pantry Clients

**DOI:** 10.1089/heq.2016.0009

**Published:** 2017-01-01

**Authors:** Jayna M. Dave, Deborah I. Thompson, Ann Svendsen-Sanchez, Karen W. Cullen

**Affiliations:** ^1^ USDA/ARS Children's Nutrition Research Center, Baylor College of Medicine, Houston, Texas.; ^2^ Department of Health and Human Performance, College of Liberal Arts and Social Sciences, University of Houston, Houston, Texas.

**Keywords:** food pantry, healthy eating, low-income

## Abstract

**Objective:** To explore perspectives on barriers of eating healthy among food pantry clients.

**Methods:** Food pantry clients participated in focus groups/interviews. Qualitative data were coded and analyzed using content analyses and grounded theory approach. Themes were then identified. Quantitative data were analyzed for frequencies and descriptives.

**Results:** Fifty-four clients from 10 pantries participated in interviews/focus groups and completed questionnaires. Two major themes emerged: concern over obesity and other chronic diseases, and barriers to healthy eating. Several subthemes for barriers to healthy eating were identified: financial uncertainty, cost of healthy foods, lack of time, rationing food within family, lack of transportation, lack of adequate kitchen equipment, lack of nutrition knowledge and skills, and social support network.

**Conclusions:** Issues identified above and those identified by others working with low-income populations need to be systematically addressed and incorporated into programs and nutrition education interventions for this group.

## Introduction

The current economic scenario in the United States has led to a historically high demand for food assistance.^1^ Unemployment and poverty rates have remained high since the Great Recession of 2008; between 2009 and 2013, the number of households receiving Supplemental Nutrition Assistance Program (SNAP) benefits increased ∼50%.^2^ In addition, the use of emergency food banks has become widespread.^2,3^

A food bank is a nonprofit organization that collects and distributes food to hunger relief charities such as food pantries. Most are primarily stocked with privately donated foods from a variety of sources such as grocers, manufacturers, wholesalers, distributors, or individuals. Although food pantry use was intended for emergencies, it is now being used to address the chronic need for food by many low-income families each month.^4^ The Houston Food Bank (HFB) is one of the largest food banks in the national network of Feeding America, the leading domestic hunger-relief charity in the United States.^4,5^ The HFB provides food assistance to more than 900,000 low-income individuals annually via 600 community-based agencies (churches, food pantries, and community kitchens).^5^

Food pantry recipients usually have low food security, poor diets, and limited access to other affordable nutritious foods.^6–8^ They often lack the knowledge, skills, and resources needed for menu planning, shopping, and food preparation.^6^ Several published studies provide insight into food pantry use and its association with food insecurity and/or nutritional status of the clients.^9^ Food pantry participation is often associated with low-income women, low education levels, single young mothers with children, and older adults living in rural areas.^10–12^ However, there is lack of research to identify the factors that influence eating behaviors and patterns among food bank clients. Therefore, the aim of this study was to explore the perceived barriers to healthy eating among low-income food bank clients to help inform future efforts to improve dietary behaviors among these high-risk individuals.

## Methods

The study was approved by the Institutional Review Board at the Baylor College of Medicine, Houston, TX. The study used interviews/focus groups to gather data on perceived barriers to healthy eating among food pantry clients.

### Sample

Participants were recruited by research staff from 10 food pantries in the Houston Metro area; all provided written consent. Eligibility included being between ages 21–50 years with at least one child (<18 years old), being the main household food preparer, and being able to talk in English. Individuals who were homeless or lived in shelters where they could not prepare their own meals were excluded. A short screener was used to assess participant eligibility.

### Data collection

This included interviews/focus groups with the participants using semistructured scripts with open-ended questions, and probes for a deeper understanding of the issues. Open-ended questions focused on factors that influence healthy eating among the participants. Participants were asked to provide insights on issues/topics such as beliefs about obesity and other chronic diseases and barriers that contribute to eating habits in their family. Each meeting was audiotaped and lasted 45–60 min. The assistant moderator took notes and recorded other relevant information (e.g., body language and facial expressions). Subsequently, the moderator and assistant moderator debriefed and discussed the main points, discrepant or new ideas that were discussed, and overall impressions.

Questions were framed in the context of Social Cognitive Theory.^13^ This theoretical framework, used widely in nutrition-related research, can help assess health behaviors and offer principles that guide behavior change.^14^ Depending on the pantry facilities and the number of clients being served, either focus groups or interviews were conducted. Our goal was to conduct interviews and/or focus groups with 60 participants or until we reached theoretical saturation. After the first few focus groups/interviews, the data were analyzed for themes. We continued to conduct further focus groups/interviews until we observed similar instances over and over again in the collected data and the themes were saturated.^15^

Before the interviews/focus groups, participants completed a pen and paper survey. This survey, available in both English and Spanish, included a demographic questionnaire; the 6-item module of the USDA household food security questionnaire^16^ that has demonstrated optimum sensitivity and specificity as well as conceptual validity^17^; a food bank usage questionnaire that assessed food bank usage frequency, types of food items obtained from the food bank, and participation in other food assistance programs (e.g., SNAP); and a home food availability checklist.^18,19^ In addition, all participants had their height and weight measured by a trained research assistant using CDC standard procedures.^20^ Body mass index (BMI) was calculated using the formula weight (kg)/height (m^2^).^21^ Note that although the survey was available in both English and Spanish, focus groups and interviews were only conducted in English.

### Data analysis

Data from the focus groups/interviews were transcribed verbatim and analyzed using the grounded theory approach, which includes open coding. Textual data were reviewed systematically. Each transcript was manually coded by two coders. The primary coder first developed a list of codes, and the secondary coder then used the list to independently code the first transcript. Preliminary codes were refined through consensus, applying codes to an additional transcript and revising the list as new concepts emerged. Proposed changes were discussed until a final consensus was reached with specified definitions for each code. The final codes were then applied to all the interviews. The inter-rater coding reliability, calculated using Cohen's kappa, was 0.87. Subsequently, axial coding was conducted during which the codes, quotations, and notes taken by the assistant moderator were added to the dominant themes.

For survey data, descriptive statistics were generated using SAS (Version 9.4). Frequency and percentages were calculated for categorical variables; means, medians, standard deviations, and ranges were used to describe continuous variables.

## Results

### Participant characteristics

Table 1 outlines the characteristics of the participants. Fifty-four participants completed the survey; mean BMI was 31.6 and the mean age was 38 years. All the participants reported being food insecure; 60% reported very low food security. About 80% were female; 44% married; 50% Hispanic; about 46% had less than high school education; about 80% reported annual household income of less than $21,000; and about 72% spoke mostly English at home. Sixty percent of the participants visited the food pantry monthly, with the remaining visiting twice a month. All participants received SNAP benefits; their children were eligible for free/reduced price school meals. Only 26% reported having fruit in the home and 42% reported vegetables; 33% reported having sugar sweetened beverages in the home and 19% reported having diet beverages; about 47% reported having whole or 2% reduced fat milk available at home and only about 13% have low-fat or fat-free milk available in the home.

**Table 1.** **Participant Characteristics (*n*=54)**

	**n**	**%**
Gender		
Male	11	20.4
Female	43	79.6
Age		
18–30	12	22.2
31–39	20	37.1
40–50	22	40.7
Ethnicity		
African American	16	29.6
White	11	20.4
Hispanic	27	50.0
Average annual household income		
<$21,000	43	79.6
$21,000–$41,000	11	20.4
Highest education		
<High school education	25	46.3
High school graduate or GED	13	24.1
>High school education	16	29.7
Marital status		
Married/living with significant other	24	44.4
Single, never married	13	33.1
Divorced, separated, or widowed	17	31.5
Food security		
Food insecurity without hunger	22	40.7
Food insecurity with hunger	32	59.3
Pantry characteristics (*n*=10)		
Church based	7	70
Community based	3	30

### Focus groups/interviews with clients

Eight focus groups with 5 participants each and 14 individual interviews (54 participants from 10 pantries) were conducted before reaching theoretical saturation. It was apparent that food choices of the participants were not solely based on food preferences, but a number of other interacting, if not constraining, personal, behavioral, and environmental factors. The themes centered around concern over obesity and other chronic diseases, and barriers to healthy eating. Themes and subthemes are listed in Table 2.

**Table 2.** **List of Themes and Subthemes**

Theme 1	Participant concern over obesity and other chronic diseases
Theme 2	Barriers to healthy eating
	Subthemes: Financial uncertainty
	Cost of healthy food
	Rationing food within the family
	Lack of time
	Lack of transportation
	Lack of adequate kitchen equipment
	Lack of nutrition knowledge and skills
	Lack of social support for eating healthy

#### Theme 1: Participant concern over obesity and other chronic diseases

Most participants believed and were concerned that obesity and other chronic diseases, especially diabetes, were a problem for them as well as their family members, including their children, and wanted to help them be healthy.

“I have high cholesterol, high blood pressure, and am border line diabetic.”

“My daughter is 2 and plump. Myself obese.”

“I don't know how I got this big. I was trying to get the surgery, but I got scared because how am I going to take care of my 2 year old. I am looking for other alternatives.”

#### Theme 2: Barriers to healthy eating

Several barriers to healthy eating were identified. These included financial uncertainty, cost of healthy food, rationing food within the family, lack of time, transportation, adequate kitchen equipment, nutrition knowledge and skills, and a social support network.

##### Financial uncertainty

Participants expressed difficulty in dealing with financial uncertainty and obtaining healthy food; they also mentioned about having to stretch dollars and felt that their SNAP benefits were not adequate to sustain throughout the month. Most participants also stated that they were concerned about being able to pay bills than the food they were eating.

“Sometimes when I am low on money I rather buy 2 pastas so they can fill up my family…..I compromise to pay the bills, water and utility is expensive too.”

“Present circumstances force me to make these difficult choices, although I would have loved to help my family eat more fruits and vegetables and milk.”

“When we get our food stamp dollars, it is like a feast. But then, especially toward the end of the month, we start checking our pockets to find you know the dollars to take care of food and pay bills.”

“With my minimum wage and three children, I find it hard to manage buying food for everyone. So I feel happy even if I am able to feed them with anything. At such times, I just want to make sure that the children have something to eat. You don't really think of eating healthy. Would you?”

##### Cost of healthy food

The cost of healthy food was identified as a barrier to eating healthy. Most participants felt that healthy foods such as fruit and vegetables tend to be more expensive. This was a consistent theme throughout all focus groups/interviews.

“It costs more to eat healthy. It is easier and wiser to buy food that we can get in bulk for cheap. At the end, it is the quantity of food that we can have….. that is when we can.”

“For us, eating different fruits and vegetables is expensive. It is hard to buy them on a regular basis since it is costly. And eating out-of-season fresh fruits and vegetables is even more expensive.”

##### Rationing food within the family

The focus groups also revealed that most parents, especially mothers, sacrificed their food when food was scarce, to be able to provide enough food to their children. Most participants used combination of stores, discount coupons, and sales to obtain food items.

“When we are almost out of food, around third week of the month, the pantry is our friend. But still I have to sometimes fast or eat very little so that I can feed my children well. I don't want them to go without food.”

“Most of the time, the food pantry is our savior.”

“I and her too, we buy food that is on sale or use those coupons from different stores. Some of those coupons are great. You can get so much with them…. Not really the healthy stuff but it helps us keep our pantry filled up so that children can eat.”

##### Lack of time

Lack of time was another barrier that the participants identified. Some of the participants worked double jobs and did not have time to cook.

“I would like to get some time to cook. But with the two jobs, I get too tired and don't have time to cook. So then my kids eat whatever is easily available at home or we go to some restaurant.”

“Both of us, my husband and I, we try to reach home in time with the hope of cooking dinner. But, the traffic is so bad that even if we leave work on time, we don't get home in time to cook. Then we just end up buying food from outside. You know like the Church's chicken bucket.”

##### Lack of transportation

About half of the participants identified lack of transportation as a barrier to eating healthy. Access to healthy foods is often limited by long distances that participants from low-income neighborhoods must travel to get to these sources.

“I don't have a car just like a couple of my other friends here…. Most of the grocery shopping we do is from the corner store and you know what we get there…Then we get food from here (pantry). We have to wait until we can get a ride to go to a decent grocery store. Sometimes it is hard for us to even get here (pantry) since I have to take two buses to get here.”

“We do not have any grocery store close by. I have to go to Fiesta in a bus. But carrying groceries on a bus is difficult, especially with children. Even to get to the pantry, I have to take two buses.”

##### Lack of adequate kitchen equipment

Few participants reported having adequate kitchen equipment such as appliances, toaster, and pots and pans. Some did not have adequate basic cooking and could not afford it either.

“I have a family of six, with me and my husband and four children years old. We do not have a blender or a toaster and only have a few pots and pans at home.”

“Our stove is a one burner stove. So its hard to cook too many things. Sometimes, we do not even have basic cooking stuff–you know like cooking oil, milk, or spices.”

##### Lack of nutrition knowledge and skills

Although older participants reported having the knowledge and skills to cook fresh food, most of the younger participants reported otherwise.

“The pantry gives us good food like rice, pasta, bread and milk. It lasts us for a few days. They also give us canned vegetables. I usually just drain them and steam them to eat. But then they also give us these fresh vegetables. I don't know how to cook with those.”

“Kids eat these steamed vegetables at school too. They don't want to eat them again at home. How do I make them more attractive for them with not much time in hand?”

“Sometimes they (food pantry) give us these fresh vegetables like spinach and carrots. I don't know what to cook with it. So I return it or they go bad in my refrigerator.”

##### Lack of social support for eating healthy

Participants reported that their children and other family members influence the food purchasing and cooking decisions that they make.

“My children are with me when I go for grocery shopping. So at the grocery store, when my children want things like chips and cookies and fried foods, and start throwing tantrums in store, I just buy them. I don't want to be embarrassed with people looking at me and my children.”

“My husband is a big meat eater. His meals are incomplete without meat. So I have to buy meat and foods that compliment meat. I then don't have enough money to buy fruits and vegetables after that.”

“My husband and now my children too, want fried foods. If it doesn't have oil, he doesn't feel comfortable. I would love to grill the chicken or meat. But they won't eat it.”

## Discussion

To our knowledge, this is the first study to identify the barriers to healthy eating among the low-income food pantry clients. There are numerous factors identified in this study that influence eating behaviors and patterns among the low-income families who obtain food from the pantries.

For many of the participants, food pantry was needed to meet their food needs. Although some may view food pantries as a plausible substitute for SNAP, food insecurity may drive motivated families to look for more sources of food assistance.^3^ All the participants in this study were food insecure and obtained food from the food pantries as an emergency food supply in addition to receiving SNAP benefits.

Prevention of obesity and related diseases may be the greatest challenge for these individuals; all participants were overweight or obese. Although health did appear to be an important factor for them, most felt it was difficult to consume a healthy diet with their dire circumstances and limited resources. Other studies with low-income individuals have found similar results.^22,23^ With the exception of a few, most participants in the current study were very frugal and had an appreciation of the importance of healthy eating. Lindsay et al. found similar results with parents being aware of the importance of healthy eating for their children.^24^ However, more research is warranted to understand how to help these individuals plan, purchase, and prepare healthy meals.

Financial uncertainty and cost of healthy foods were identified as the most significant barriers to healthy eating. Participants felt that healthy eating was not possible given that they lived on limited resources. An older study also found that pantry users were more likely to report having difficulty adequately feeding their families, running out of money to buy food, serving less nutritive foods to stretch their budget, and borrowing money from friends and family for food.^10^ The perception that healthy foods are more expensive than unhealthier options supports the reported low amounts of fruit and vegetables available in the home. Cost has been found to be a significant barrier to healthy eating in several other published studies with low-income participants.^24–26^

Purchasing and preparing a healthy diet require time. American cooking habits in the past few decades reflect the effects of hectic work and home schedules. The number of hours worked has increased, especially among low-income families; adults sometime report double jobs. Another study has also found similar results with Latino parents reporting lack of time due to competing daily demands, including work obligations and long workdays.^24^ Time spent on meal preparation has continued to drop.^27^ These changes have worked against home food preparation and encouraged purchase of convenience and restaurant foods. In a cross-sectional study with more than 3,700 diverse parents of adolescents, mothers working full time reported spending less time in meal preparation, preparing fewer family meals, and consuming fewer fruits and vegetables.^28^

In this study, some participants mentioned the stress of living in poverty and how they compromised their food to ensure their children had something to eat, thus compromising their nutritional status. Similar findings were reported among low-income Canadian mothers, who consumed diets consistently lower in nutrients compared to their children, over a one-month period.^29,30^ This phenomenon, referred to as “maternal deprivation,” is paradoxical considering the high rate of overweight and obesity in the sample. However, this phenomenon may be partially explained by an eating cycle of binging on inexpensive, calorie-dense foods when available, following a period of food scarcity.^31^

The availability of personal, nonpublic transportation is an additional factor influencing food choice among low-income families. Households without a personal vehicle or access to public transportation rely on alternative means—walking or biking to a store close at hand or using family and/or friends to provide transport. While the issue of transportation is a complicated one, car ownership appears to be the most important correlate of pantry use among low-income households.^10^ An alternative is innovative localized food distribution systems. One example is mobile pantries to take food to areas for distribution.^10^

Lack of adequate kitchen equipment was another barrier. Low-income families may lack much of the equipment and ingredients needed to prepare fresh foods. Major appliances might be absent or be inadequate for storage and food preparation. Some low-income individuals live with friends or family members or in rooming houses where they may not have access to proper food storage or preparation space.

Lack of nutrition knowledge and skills also influences food choices and is not uncommon in low-income households.^32^ Because a healthy diet is one of the major components of a healthy lifestyle, increasing nutrition knowledge and skills in menu planning, grocery shopping, and food preparation is crucial. However, a comprehensive nutrition education intervention must target these skills and knowledge for making the right food choices and also match the client living circumstances.

One important finding of the study is the influence of the social support network on food choices and consumption. Parents play an important role in developing children's dietary habits; the foods made available and eaten by parents are those that become familiar and acceptable to children.^33^ Children influence the purchasing decisions of their parents, but this influence decreases when parents become more aware of the importance of a healthy diet.^34^

Although the results from this study add richness and depth to the current body of literature, findings may not be representative of all low-income food bank clients. The clients were from one geographic location, limiting generalizability. In addition, limiting participants to English speakers limits our ability to uncover experiences among pantry users from different cultures. Although the sample size for food bank clients was small, we did obtain theoretical saturation, which makes this less of a concern. Another limitation of the study was that since during coding and analysis the quotes were interspersed from the focus groups and interviews, identifying if the quote is from focus group or interview was not possible. Also, all personal information was deidentified during the focus groups, and thus, it was not possible to note variations in individual or personal characteristics of the participant (ethnicity/age) with the quote.

## Conclusion

Issues identified above and those identified by others working with low-income populations need to be systematically addressed and incorporated into programs and nutrition education interventions for this group. A detailed perspective of the household context is essential for both the nutrition educators/researchers and families themselves for an improvement in nutritional health of low-income families and their children.

Examining the trigger events in people's lives that cause them to start using food pantries could be fruitful. Not all low-income people use food pantries. Understanding what leads some and not others to use food pantries would allow for appropriate programs and policies to be developed.

## Abbreviations Used

HFB: Houston Food Bank
SNAP: Supplemental Nutrition Assistance Program
